# 16S rRNA seq-identified Corynebacterium promotes pyroptosis to aggravate diabetic foot ulcer

**DOI:** 10.1186/s12879-024-09235-x

**Published:** 2024-04-01

**Authors:** Hailong Zheng, Han Na, Jiangling Yao, Sheng Su, Feng Han, Xiaoyan Li, Xiaopan Chen

**Affiliations:** 1 Department of Endocrinology, The First Affiliated Hospital of Hainan Medical University, Hainan Province, No. 31, Longhua Road, Haikou City, 570102 China; 2 Department of Wound Repair, The First Affiliated Hospital of Hainan Medical University, Hainan Province, No. 31, Longhua Road, Haikou City, 570102 China; 3 Department of Clinical Laboratory, The First Affiliated Hospital of Hainan Medical University, Hainan Province, No. 31, Longhua Road, Haikou City, 570102 China

**Keywords:** 16S sRNA sequencing, Bacterial infection, Corynebacterium, Diabetic foot ulcer, Immune

## Abstract

**Background:**

Diabetic foot ulcer (DFU) is one of the main chronic complications caused by diabetes, leading to amputation in severe cases. Bacterial infection affects the wound healing in DFU.

**Methods:**

DFU patients who met the criteria were selected, and the clinical data were recorded in detail. The pus exudate from the patient’s foot wound and venous blood were collected for biochemical analysis. The distribution of bacterial flora in pus exudates of patients was analyzed by 16S rRNA sequencing, and the correlation between DFU and pathogenic variables, pyroptosis and immunity was analyzed by statistical analysis. Then, the effects of key bacteria on the inflammation, proliferation, apoptosis, and pyroptosis of polymorphonuclear leukocytes were investigated by ELISA, CCK-8, flow cytometry, RT-qPCR and western blot.

**Results:**

Clinical data analysis showed that Wagner score was positively correlated with the level of inflammatory factors, and there was high CD3^+^, CD4^+^, and low CD8^+^ levels in DFU patients with high Wagner score. Through alpha, beta diversity analysis and species composition analysis, Corynebacterium accounted for a large proportion in DFU. Logistics regression model and Person correlation analysis demonstrated that mixed bacterial infections could aggravate foot ulcer, and the number of bacteria was closely related to inflammatory factors PCT, PRT, immune cells CD8^+^, and pyroptosis-related proteins GSDMD and NLRP3. Through in vitro experiments, Corynebacterium inhibited cell proliferation, promoted inflammation (TNF-α, PCT, CRP), apoptosis and pyroptosis (IL-1β, LDH, IL-18, GSDMD, NLRP3, and caspase-3).

**Conclusion:**

Mixed bacterial infections exacerbate DFU progression with a high predominance of Corynebacterium, and Corynebacterium promotes inflammation, apoptosis and pyroptosis to inhibit DFU healing.

**Supplementary Information:**

The online version contains supplementary material available at 10.1186/s12879-024-09235-x.

## Introduction

Diabetic foot ulcer (DFU) is one of the main chronic complications caused by diabetes, which is non-healing wound caused by microangiopathy, diabetic neuropathy, wound bacterial infections, and other vascular diseases [1]. In severe cases, it will lead to lower limb amputation and death [2, 3]. Studies have shown that the 5-year mortality rate of DFU is up to 30% [4], which has become a global medical problem. Therefore, early diagnosis and prevention of diabetic foot complications is very important. At present, the treatments for foot ulcers mainly includes surgical debridement, reduction of foot weighing pressure, and treatment of lower limb ischemia and foot infection [4]. Some smart wound dressings are applied in the wound healing, such as 3D-printed wound dressings, smart and flexible bandages, and biomolecule-loaded dressings [5]. In addition, negative pressure wound therapy combined with platelet-rich plasma therapy has a significant effect in the treatment of DFU [6]. It is of great significance to understand the factors affecting DFU wound healing for the treatment of the disease.

Wound healing is achieved through four stages: hemostasis, inflammation, proliferation, and remodeling, and it mainly affected by infection, oxygenation, and systemic factors such as hormones, diabetes, and drugs [7]. When the skin is injured, the microorganisms on the skin surface will enter the body from the wound, prolonging the inflammatory period and resulting in prolonged wound healing time or even failure to heal. It is reported that bacteria on the skin surface play a significant role in the pathology and physiology of DFU, which may be detrimental to wound healing [8]. In DFU, Staphylococcus, Corynebacterium, Pseudomonas, and several anaerobes are observed in wound [9]. It has been reported that inflammation is closely related to pyroptosis, which can promote inflammation and thus promote tissue damage [10]. Pyroptosis is a programmed cell death triggered by inflammasomes that activate caspase family proteins, which lead to cleavage of the gasdermin protein, disrupting the osmotic barrier of the plasma membrane and causing cell rupture [11, 12]. Previous studies have demonstrated the important role of pyroptosis in diabetic wound healing. Major mediators of pyroptosis (NLRP-3, caspase-1, and IL-1β) are highly expressed in diabetic wounds [13]. In DFU, intracellular *Staphylococcus aureus* causes pyroptosis, which prolongs the inflammatory period and inhibits wound healing [14].

16S ribosomal RNA (16S rRNA) sequencing is a method for identifying bacteria in clinical microbiology and infectious diseases [15], and is widely used in many fields. 16S rRNA gene targeted metagenomic sequencing is used to detect pathogenic bacteria in patients with sepsis [16]. Wang et al. sequence the microbiome in the blood of patients with polycystic ovary syndrome by 16S rRNA to explore its relationship with the disease [17]. High-throughput sequencing of the 16S rRNA can also analyze the gut microbiota that characterizes autism spectrum disorders [18]. 16S rRNA is a rapid microbiological diagnostic method for DFU, which takes less time [19]. Therefore, it is of vital importance to carry out the microflora analysis in DFU through 16S rRNA sequencing.

In this study, DFU patients were selected and their pathological data were collected for analysis. Through 16 s rRNA sequencing analysis of the patients’ foot wound secretions, the microflora species affecting wound healing were explored, and the relationship between the microflora and various pathogenic factors was examined by statistical analysis. It was found that Corynebacterium was widely distributed in wound. In vitro experiments demonstrated that Corynebacterium could significantly inhibit cell proliferation, promote apoptosis and pyroptosis.

## Materials and methods

### Clinical samples

#### Inclusion criteria

(1) Patients who met the diagnostic criteria for diabetes (using oral antidiabetics only, except patients with high HbA1c levels) and were hospitalized with foot ulcers. (2) The patients have been sent to hospital for drug sensitivity test and bacterial culture. (3) According to the Wagner classification, it belongs to grade 0 ~ 5; (4) The clinical data of the patients were complete, including drug sensitivity test, bacterial culture experiment, blood glucose, blood lipid, ankle-brachial index, oxygen partial pressure and other baseline data. (5) Patients were informed of this study and signed informed consent.

#### Exclusion criteria

Patients: (1) Recent use of hyperbaric oxygen therapy. (2) Diagnosed with severe liver and kidney dysfunction. (3) Diagnosed with tumors, autoimmune disease, or diseases of blood system. (4) Diagnosed with chronic venous disease. (5) Lack of clinical data or voluntary application to withdraw from the study.

#### Case collection

Twenty-four DFU patients who met the criteria and were admitted between June 2021 and October 2023 were clinically screened as the study subjects. The patients’ data were recorded in detail, including gender, age, duration of diabetes, degree of foot ulcer, Wagner classification, blood glucose, blood lipids, liver function and renal function indicators. Specimens were collected from all enrolled patients at the time of the first foot wound dressing change, and pus or secretions were wiped from superficial ulcerated wounds using sterilized saline cotton swabs. Deep pus exudates were collected after debridement of necrotic tissue and deep ulcerated wounds, preserved in sterilized test tubes, and promptly sent for examination. Fasting venous blood was collected in the first morning of the day after clinical screening.

This study was approved by the Medical Ethics Committee of the First Affiliated Hospital of Hainan Medical University.

### Enzyme linked immunosorbent assay (ELISA)

Following the manufacturer’s instructions, the levels of inflammatory factors (CRP, PCT, TNF-α), immune cells CD3^+^, CD4^+^, and CD8^+^, and the expression of IL-1β, LDH, and IL-18 in the supernatant were measured using specific ELISA kits (Esebio, Shanghai, China).

### Real-time fluorescent quantitative PCR (RT-qPCR)

Total RNA was extracted from tissues using TRIzol (Invitrogen, USA). The obtained RNA was dissolved by DEPC water, and 2 μL of RNA was taken to determine the concentration of RNA on the Nano-100. cDNA was synthesized using the Hiscript II QRT Supermix for qPCR (Vazyme, Nanjing, China). The reaction conditions were: 50 °C, 15 min and 85 °C, 5 s. RT-qPCR was performed using ChamQ SYBR qPCR Master Mix (Vazyme). The conditions were: 95℃ pre-denaturation for 30 s, 40 cycles (95℃, 10 s, 60℃, 30 s, and 95℃, 15 s), followed by 60℃ for 60 s. GAPDH was set as the internal control. The obtained Ct value was analyzed by 2^−ΔΔCt^ method, and the experiment was repeated three times. Primer sequences are shown in Table S1.

### 16S rRNA sequencing

DFU pus samples were subjected to 16S rRNA sequencing to find the key pathways and flora composition that affect wound healing. Logistics regression analysis and Person correlation analysis were performed afterwards.

### Cell culture

Polymorphonuclear leukocytes (PMNL) were cultured in DMEM (HyClone, Cytiva, USA) containing 10% fetal bovine serum (Biosera, France) and 1% penicillin–streptomycin (Beyotime, Shanghai, China). All cells were cultured at 37℃, 5% CO_2_ and 95% humidity. After culture for one week, the cells were passaged and divided into the Control group and Corynebacterium group (PMNL were co-cultured with *Corynebacterium striatum*).

### Cell Counting Kit-8 (CCK-8) assay

CCK-8 assay was performed to detect the effect of *Corynebacterium striatum* on PMNL cell viability. PMNL (1 × 10^4^/well) in each group were seeded into 96-well plates at 37℃, 5% CO_2_ for 24 h. CCK-8 solution (Solarbio, Beijing, China) was added into each well at 24, 48, 72 and 96 h after treatment. The plates were incubated for 2 h at 37 °C. At last, OD_450_ was measured by a microplate reader (Thermo Fisher scientific, Waltham, MA, USA).

### Flow cytometry

Flow cytometry was used to detect the effect of *Corynebacterium striatum* on PMNL apoptosis. The apoptosis of PMNL was detected according to the instructions. The cells were collected and resuspended with FITC-labeled Annexin V and propidium iodide (Solarbio). After incubation at room temperature for 15 min, the cells were diluted with binding buffer and analyzed by flow cytometry.

### Western blotting

Western blotting was executed to measure the effect of *Corynebacterium striatum* on pyroptosis. RIPA lysis buffer (Beyotime, Nanjing, China) was used to extract proteins from cells. Proteins were separated by electrophoresis on 10% SDS-PAGE and transferred to a PVDF membrane. Then they were incubated with primary antibodies of anti-GAPDH (1:1000, ab9485, Abcam, Cambridge, UK), anti-NLRP3 (1:1000, ab263899, Abcam), anti-GSDMD (1:1000, ab219800, Abcam) and anti-caspase-3 (1:1000, ab32351, Abcam) at 4 °C overnight followed by goat anti-rabbit secondary antibody (1:5000, ab6721, Abcam) for 2 h at room temperature. After treatment of enhanced ECL Chemiluminescence Detection Kit (Vazyme), the membrane was plated and exposed on Tanon 5200 Chemiluminescent Imaging System Incubation Plate. The gray value of proteins was calculated by Image J.

### Statistical analysis

#### 16S rRNA sequencing analysis

Raw data were removed from low-quality sequences, and problematic samples were retested and patch-tested. Raw sequences that passed the initial quality screening were divided into libraries and samples according to index and barcode information, and barcode sequences were removed. Sequence denoising or OTU clustering was performed according to the QIIME2 DADA2 analysis process or the analysis process of Vsearch software. The specific composition of each sample at different species taxonomic levels was demonstrated.

Based on the distribution of ASV/OTU in different samples, the Alpha diversity level of each sample was assessed and the appropriateness of sequencing depth was reflected by the sparse curve. The distance matrix of each sample is calculated, and the beta diversity difference between different samples was measured by a variety of unsupervised sorting and clustering methods, combined with the corresponding statistical test methods.

Through various unsupervised and supervised sorting, clustering, and modeling methods, combined with corresponding statistical test methods, the differences in species abundance composition among different samples were further measured, and the marker species were found. According to the composition and distribution of species in each sample, the association network was constructed, the topological index was calculated, and the key species were identified. According to the results of 16S rRNA gene sequencing, the metabolic function of the samples can be predicted, the differential pathways can be found, and the species composition of specific pathways can be obtained.

#### Logistics regression analysis

Binary Logistics in IBM SPSS 21 was performed to analyze the relationship between the distribution of bacterial microflora in foot ulcer wound secretions of DFU patients obtained by 16S rRNA sequencing and the pathogenic variables of patients (Infection with two or more genera is considered as mixed bacterial infection), including gender, age, inflammatory factors (TNF, CRP, PCT), immune cells (CD3^+^, CD4^+^, CD8^+^) and Wagner classification. The disease-related regression equation was generated.

#### Person correlation analysis

The Person correlation analysis in IBM SPSS 21 was used to determine the correlation among various pathogens present in DFU patients, pyroptosis and immunity. The two-sided test was selected for significance test.

Data were processed by GraphPad Prism 9.0 and expressed as multiple sets of repeated data or means ± SD. One-way ANOVA and Tukey’s test were used for comparison between multiple groups. The t-test was performed for comparison between the two groups, and *P* < 0.05 was regarded statistically significant.

## Results

### The expression of inflammatory factors and immune cells in clinical DFU

**Fig. 1:**
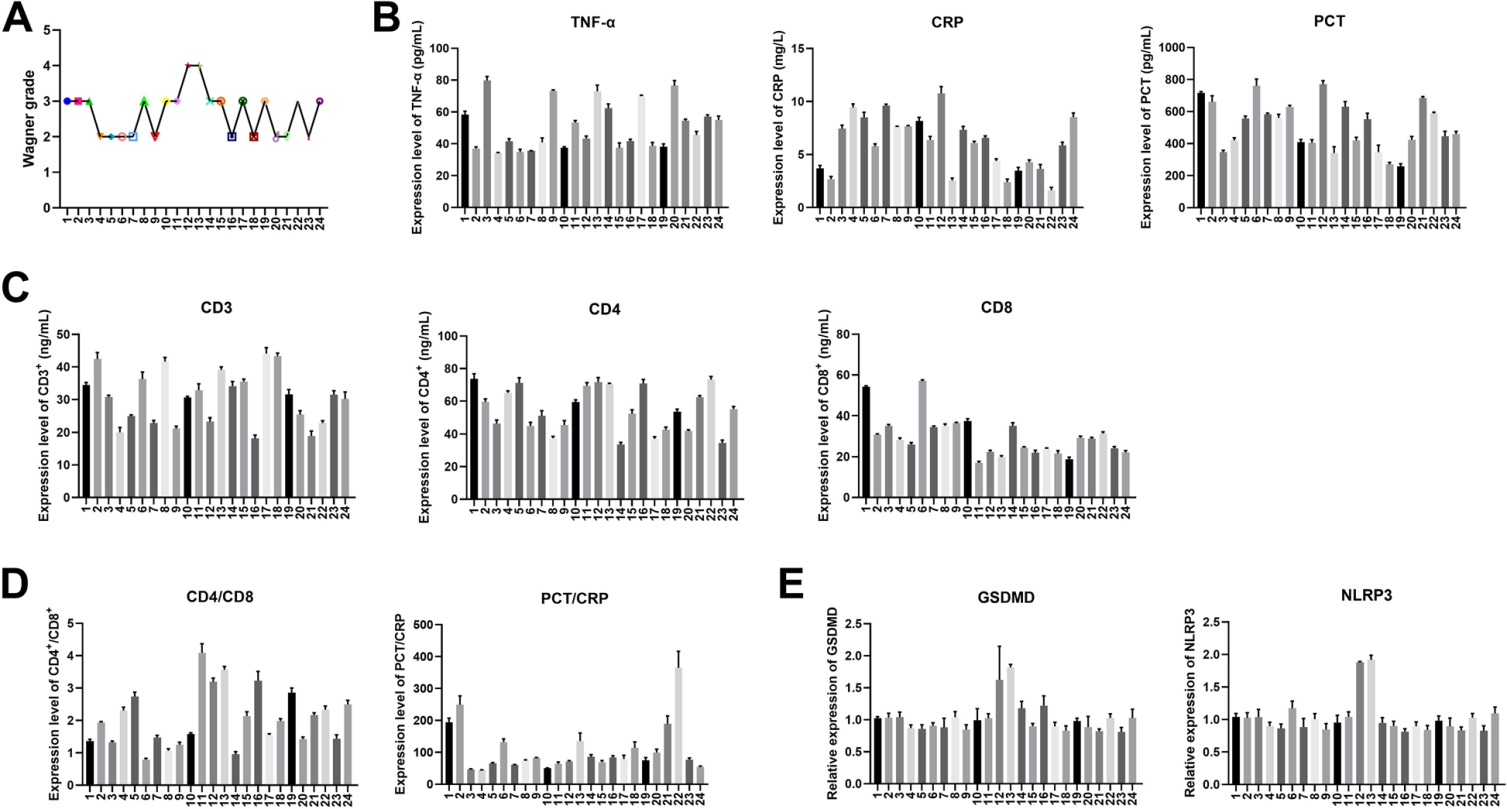
**A** Wagner classification of 24 clinical patients with diabetic foot disease. **B** ELISA was used to detect the levels of serum TNF-α, CRP and PCT. **C** ELISA was performed to detect the expression of CD3 + , CD4 + and CD8 + in serum. **D** The ratio of CD4 + /CD8 + and PCT/CRP was calculated. **E** RT-qPCR was used to detect the relative expression of GSDMD and NLRP3

The Wagner score was performed on the affected feet of 24 patients with diabetic foot disease in the clinic, showing that 22 patients’ scores were in the range of 2–3, and only two patients’ scores reached 4 (Fig. 1A). The inflammatory factors and immune cell levels of the patients’ fasting blood were examined using ELISA, and combined with the Wagner score, it was found that the patients’ Wagner score was positively correlated with their serum inflammatory factor expression levels (Fig. 1B). Patients with high Wagner scores had higher serum levels of CD3^+^ and CD4^+^, while CD8^+^ was reduced (Fig. 1C). The values of CD4^+^/CD8^+^ and PCT/CRP were calculated, indicating that CD4^+^/CD8^+^ was increased in patients with high Wagner score (Fig. 1D). RT-qPCR demonstrated that the levels of GSMDM and NLRP3 were relatively higher in patients with high foot scores (Fig. 1E). The blood biochemical indexes of lipids, blood glucose, liver function and kidney function were analyzed in each patient (Table 1).

**Table 1:** Analysis of blood biochemical indexes in patients with diabetic foot

Blood biochemical indexes	Categories	Mean ± Standard Deviation
Blood lipid	Total Cholesterol (mmol/L)	3.91 ± 1.31
	Triglycerides (mmol/L)	1.45 ± 0.71
	High-density lipoprotein cholesterol (mmol/L)	0.82 ± 0.27
	Low-density lipoprotein cholesterol (mmol/L)	2.30 ± 0.88
Liver function	Alanine aminotransferase (U/L)	20.78 ± 34.89
	Aspartate aminotransferase (U/L)	22.51 ± 21.25
Renal function	Creatinine (μmol/L)	127.94 ± 163.72
	Urea nitrogen (mmol/L)	8.08 ± 5.51
	Total protein (g/L)	62.41 ± 6.48
	Albumin (g/L)	24.80 ± 5.46
Blood glucose	Glycated hemoglobin (%)	8.44 ± 2.33
	Blood glucose (mmol/L)	6.87 ± 3.69

### 16S rRNA sequencing analysis

#### Alpha diversity analysis

**Fig. 2:**
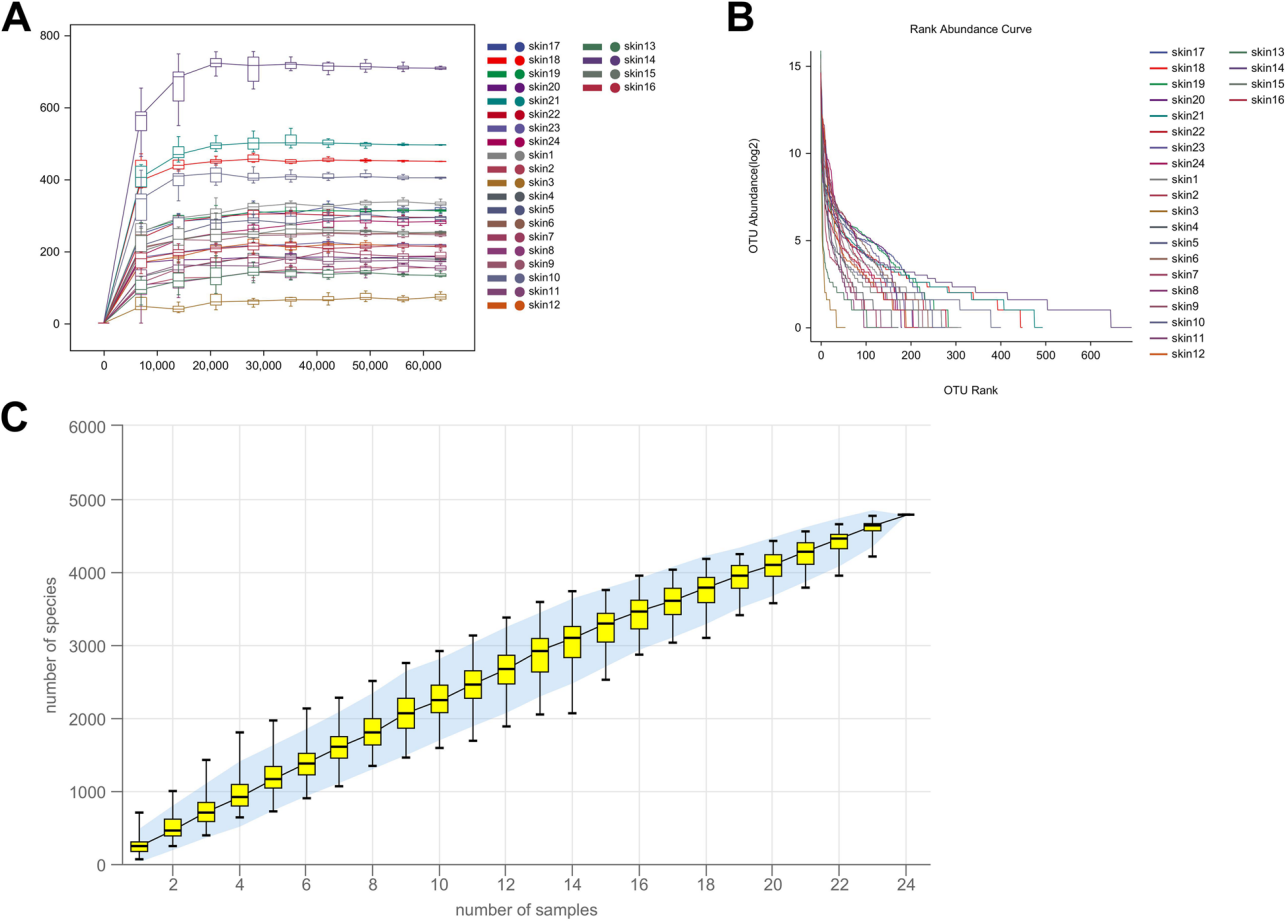
Alpha diversity analysis. **A** Sparse curve. **B** Abundance grade curve. The richness of species is reflected by the length of the curve on the horizontal axis. The wider the curve, the richer the species composition. The uniformity of species composition is reflected by the shape of the curve. The more flat the curve is, the higher the uniformity of species composition is. The smoother the curve is, the higher the species diversity of the sample is, and the rapid and steep decline of the curve indicates that the proportion of dominant bacteria in the sample is high and the diversity is low. **C** Species accumulation curve

Alpha diversity refers to the diversity in a specific area or ecosystem, which is a comprehensive index reflecting richness and evenness. In the sparse curve, the curve of No.20 patient was the highest, indicating the most abundant species composition and the highest diversity at the same sequencing depth. The number of species from high to low was No.21, No.18, and No.23 (Fig. 2A). The curve of No.3 group decreased the fastest with a narrow width, indicating that the species composition was not rich and the diversity was low (Fig. 2B). The species accumulation curve was used to measure and predict the increase of species richness in the community with the increase of sample size. The total number of ASV/OTU in the community will no longer increase significantly with the addition of new samples, and the curve tended to be gentle (Fig. 2C).

#### Beta diversity analysis

**Fig. 3:**
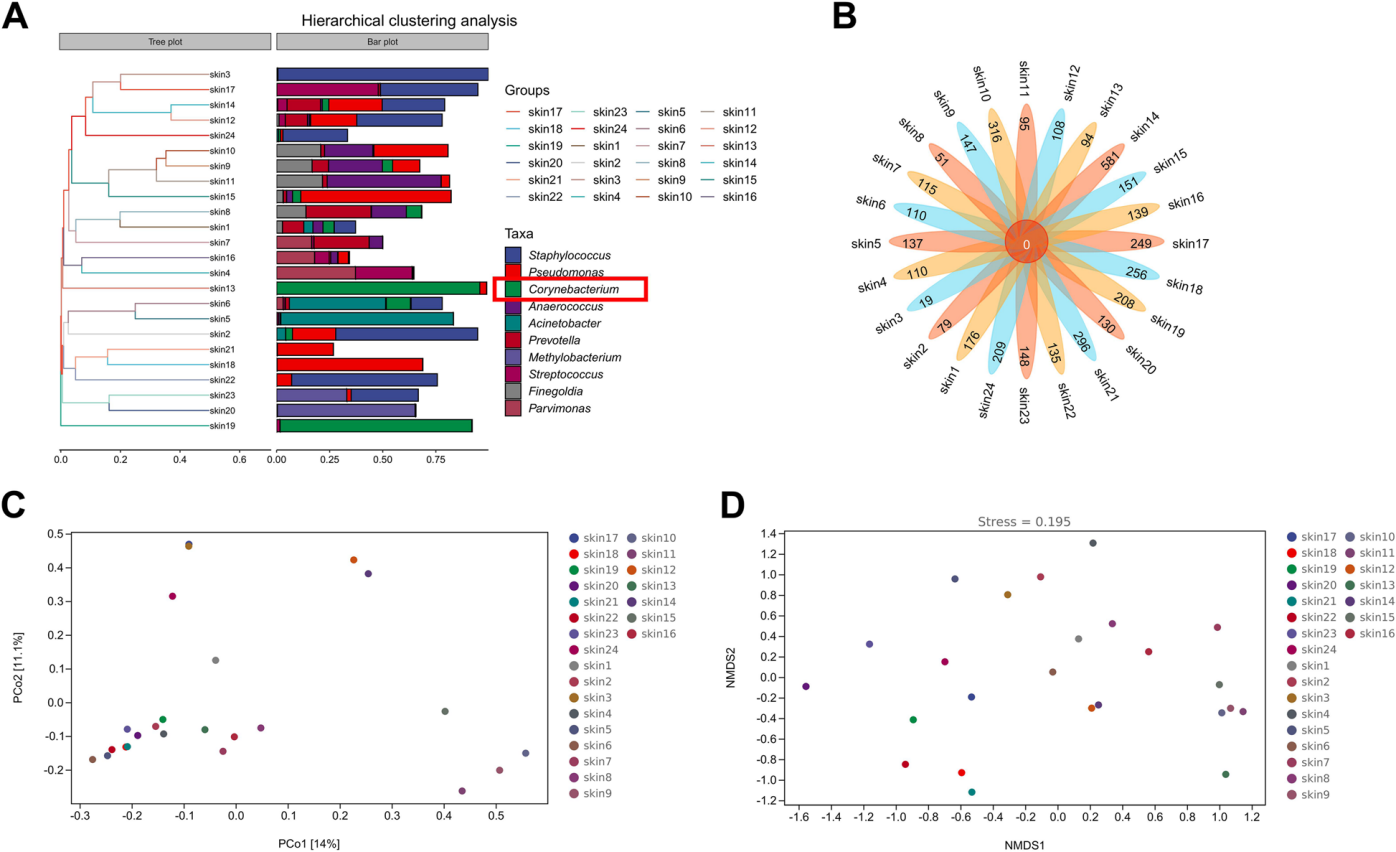
Beta diversity analysis. **A** Hierarchical cluster analysis. The shorter the branch length between samples is, the more similar the two samples are. **B** Petal plot of species difference analysis. The common species and unique species between different sample groups were analyzed. **C** Distance matrix and PCoA analysis chart. Each point in the figure represents a sample, and points of different colors indicate different groups. The percentage in the coordinate axis brackets represents the proportion of sample difference data (distance matrix) that the corresponding coordinate axis can explain. **D **Non-metric multidimensional scaling (NMDS) analysis

Hierarchical clustering analysis showed the similarity between samples in the form of hierarchical tree, and measured the clustering effect by the branch length of clustering tree (Fig. 3A). Petal plots were used for community analysis to analyze common species and unique species between different sample groups. The results showed that the number of microbial species shared by 24 patients was 0, and the number of specific species was different (Fig. 3B). The distance matrix and PCoA analysis were carried out. The distance between the sample points in the space can reflect the sample difference distance in the distance matrix to the greatest extent. The closer the projection distance of the two points on the coordinate axis was, the more similar the community composition of the two samples in the corresponding dimension was (Fig. 3C). Non-measured multidimensional scaling (NMDS) analysis allowed samples to be ranked in low-dimensional space to match as closely as possible the proximity of similar distances to each other (rather than exact distance values) by ranking sample distances in a hierarchical order (Fig. 3D).

#### Species distribution and species composition analysis of metabolic pathways

**Fig. 4:**
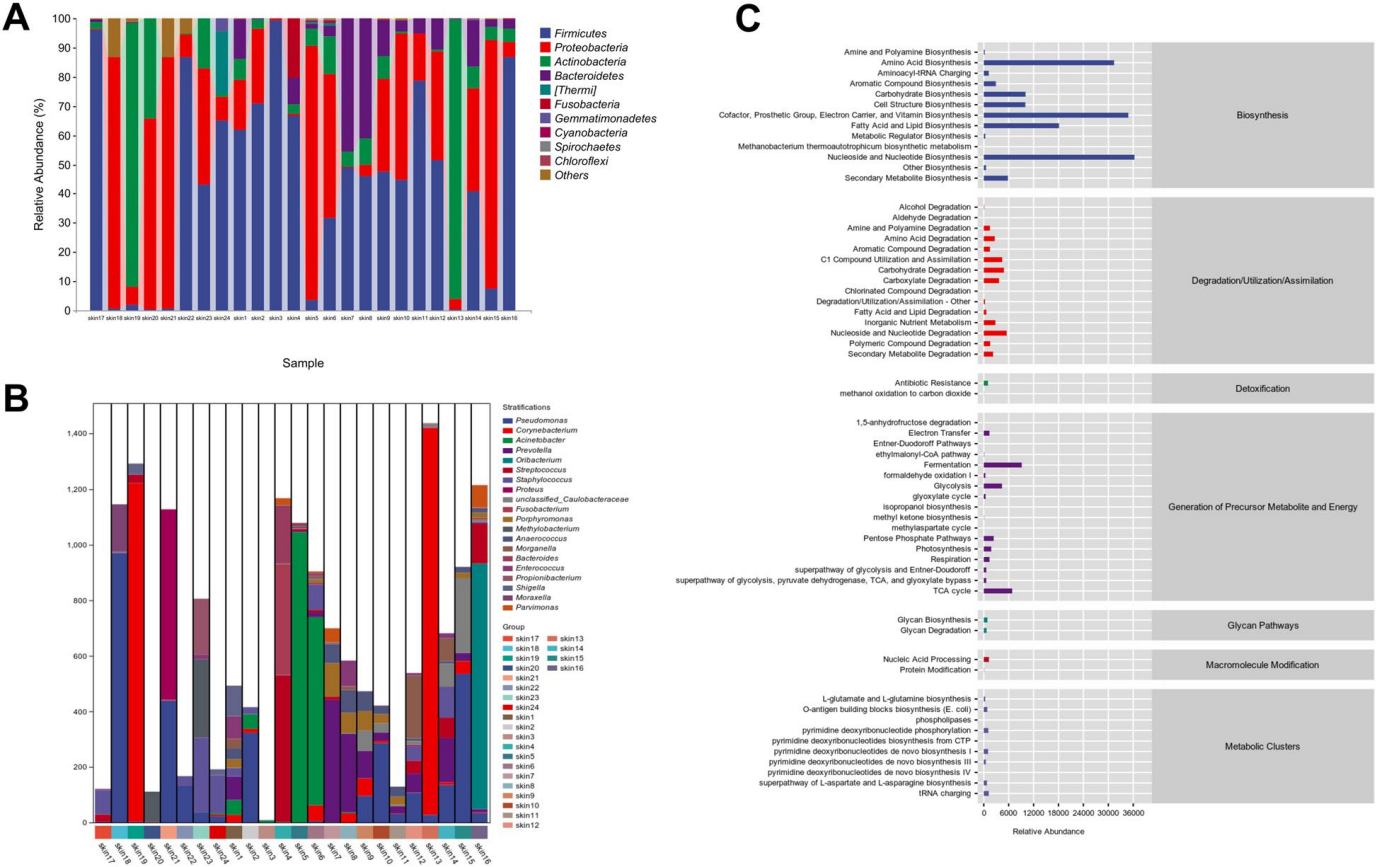
**A** Analysis of taxonomic composition. **B** Species composition map of metabolic pathways. **C** Metabolic pathway statistics

Taxonomic composition analysis realized the visualization of the composition distribution of each sample at the six classification levels of phylum, class, order, family, genus, and species by counting the feature table after removing singleton, and presents the analysis results with a histogram (Fig. 4A). The species composition of the metabolic pathway was analyzed, showing that Corynebacterium accounted for a large proportion of the species composition of the metabolic pathway except for unclassified strains. Pseudomonas and Acinetobacter accounted for a large proportion and participated in the main metabolism (Fig. 4B). According to the statistics of related metabolic pathways, Nucleoside and Nucleotide Biosynthesis accounted for the largest proportion in biosynthesis (Fig. 4C).

### Influence factors of mixed bacterial infection in DFU patients analyzed by Logistics regression model

As shown in Table 2, most DFU patients had mixed bacterial infection. The Wagner score was higher in the patients with mixed bacterial infection, and the expression of PCT was significantly higher than that of non-mixed bacterial patients.

**Table 2:** Statistics on the relationship between mixed bacteria and influence factors in diabetic foot disease

Clinical indicator		Diabetic foot disease with mixed bacterial infection (Yes/No)			
		**Yes**	**No**	**t**	***P***
Gender	Male	13	1	0.06	0.807
	Female	9	1		
Age (years old)		62.68 ± 11.05	65.5 ± 24.75	3.884	0.061
TNF		51.1984 ± 15.31524	46.5159 ± 11.21	1.149	0.295
CRP		8.1986 ± 1.90	7.355 ± 1.00	1.53	0.229
PCT		593.3294 ± 145.53	527.3622 ± 60.52	4.474	< 0.05
CD3		30.628 ± 7.54	31.0817 ± 17.31	4.005	0.058
CD4		55.3508 ± 13.83	52.52 ± 13.92	0.186	0.67
CD8		27.9846 ± 6.96	25.1947 ± 5.11	1.369	0.255
Wagner grade		2.73 ± 0.63	2	5.108	< 0.05

Through Logistics regression model analysis, the Logistic regression formula of the pathogenic factors related to the occurrence of mixed bacterial infection in DFU was obtained as follows (Table 3): Ln((P/(1-P)) = 10.62*Age + 12.709*TNF-α + 15.584*CRP + 11.38*PCT + 19.781*CD3- + 17.385*CD4 + 14.158*CD8 + 11.394*Wagner-14.31.

**Table 3:** Variables in Logistics regression model

Variables in the equation							
	**Variables**	**B**	**S.E,**	**Wals**	**df**	**Sig**	**Exp (B)**
**Step 4**	Age	10.62	1.279	43.695	1	0.008	4.538
	TNF-α	12.709	5.343	44.076	1	0.003	5.067
	CRP	15.584	4.245	23.727	1	0.002	5.025
	PCT	11.38	5.136	22.091	1	0.018	3.976
	CD3 +	19.781	2.917	31.561	1	0.018	4.146
	CD4 +	17.385	4.497	41.289	1	0.004	5.301
	CD8 +	14.158	1.953	28.259	1	0.009	1.567
	Wagner grade	11.394	0.429	16.037	1	0.009	2.256
	Constant	-14.31	13.606	18.952	1	0.000	0.445

### Correlation study of bacterial infection and immune function in DFU patients by Person correlation analysis

Person correlation analysis showed that the number of infected strains in the affected area was closely related to the inflammatory factors PCT and CRP, the immune cells CD8^+^, and pyroptosis-related targets GSDMD and NLRP3 (Table 4).

**Table 4:** Person correlation analysis of the relationship between the number of bacteria in the affected feet and the related factors

		Inflammatory factors			Immune cells			Pyroptosis-related targets	
		PCT	CRP	TNF-α	CD3 +	CD4 +	CD8 +	GSDMD	NLRP3
Number of mixed strains	Pearson correlation	0.644	0.539	0.182	0.109	0.089	0.519	0.493	0.504
	Significance (two-sided)	0.001	0.007	0.395	0.611	0.679	0.009	0.014	0.012

In the clinical data, we have found that CD8^+^ level was decreased and CD4^+^/CD8^+^ ratios were abnormally increased in DFU patients with high Wagner scores (Fig. 1C-D). Combined with Person correlation analysis, it was concluded that bacterial infection was significantly associated with immune function in DFU patients.

### Corynebacterium inhibits cell proliferation, promotes inflammation and apoptosis

**Fig. 5:**
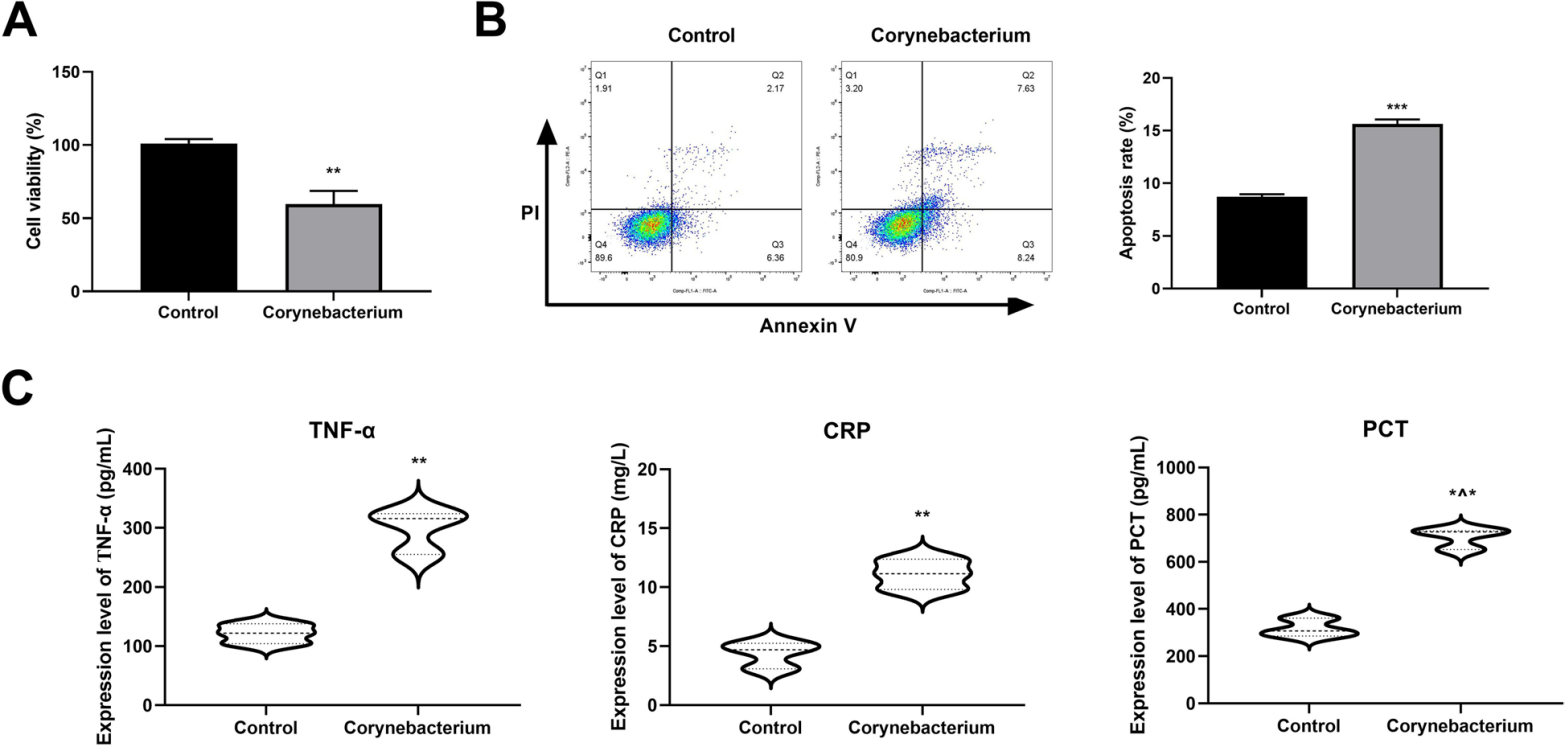
**A** CCK-8 was used to detect cell viability. **B** Apoptosis was detected by flow cytometry. **C** The levels of inflammatory factors TNF-α, PCT and CRP in cells were measured by ELISA. ^**^P < 0.01 ^***^P < 0.001 *vs*. Control

The effects of Corynebacterium on DFU were carried out in vitro. The cell viability was detected by CCK-8 method, demonstrating that compared with the Control group, Corynebacterium significantly reduced the cell viability (Fig. 5A). Flow cytometry was performed to detect apoptosis, which showed that compared with the Control group, the apoptosis rate in the Corynebacterium group was significantly increased (Fig. 5B). ELISA showed that compared with the Control group, the levels of inflammatory factors (TNF-α, PCT, and CRP) in the Corynebacterium group were significantly increased (Fig. 5C).

### Corynebacterium promotes pyroptosis

**Fig. 6:**
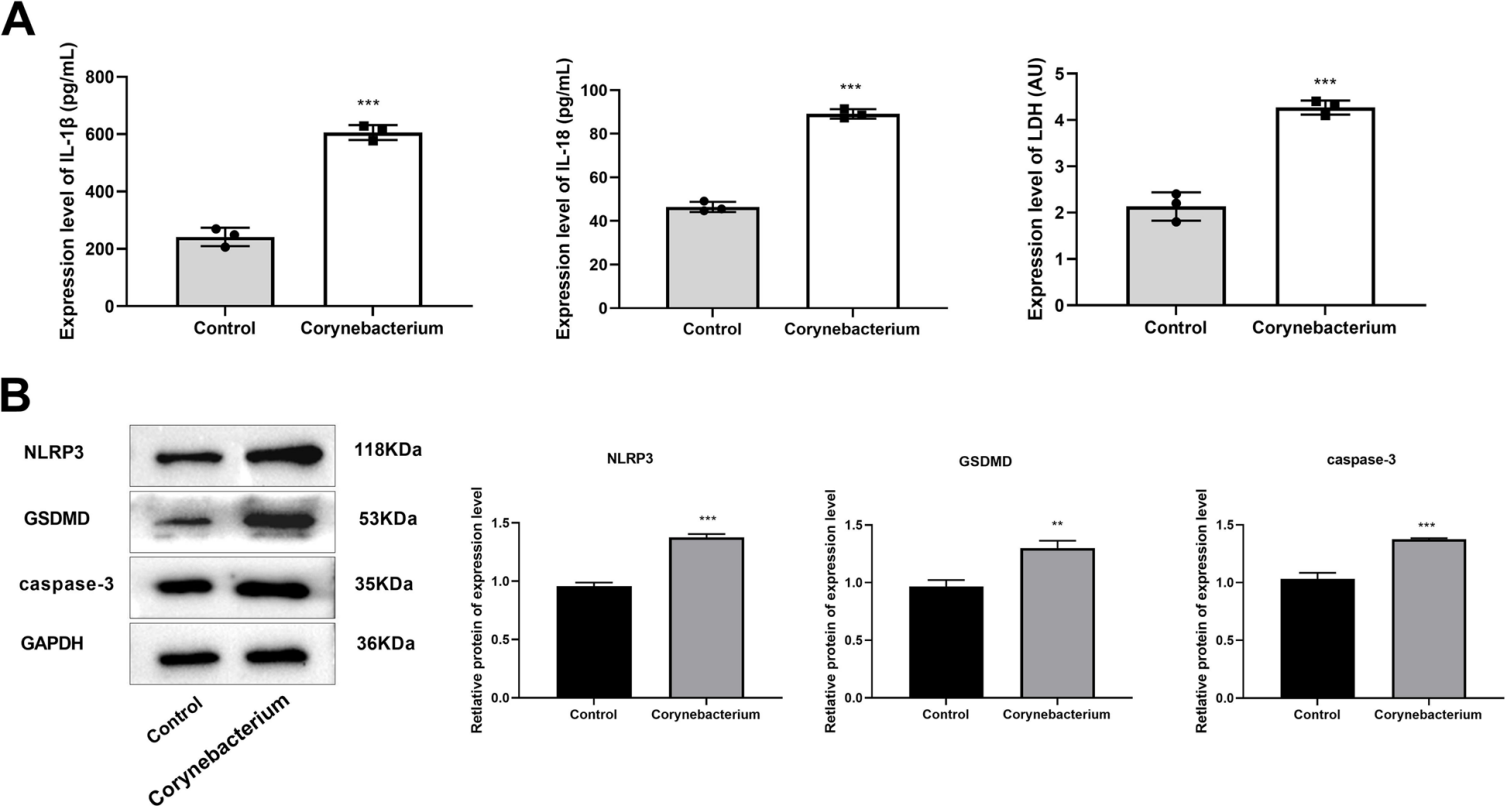
**A** ELISA was carried out to determine the levels of IL-1β, LDH and IL-18 in cells. **B** Western blot was performed to determine the expression of pyroptosis-related proteins (GSDMD, NLRP3 and caspase-3) in cells. ^**^P < 0.01 ^***^P < 0.001 *vs*. Control

The levels of pyroptosis-related pro-inflammatory factors IL-1β, IL-18, and LDH in the cells were measured by ELISA. Compared with the Control group, the levels of IL-1β, LDH and IL-18 in Corynebacterium group were significantly increased (Fig. 6A). Western blot was used to determine the expression of pyroptosis-related proteins, revealing that compared with the Control group, the relative expression of GSDMD, NLRP3, and caspase-3 in the Corynebacterium group was significantly increased (Fig. 6B).

## Discussion

DFU is the most common foot injury leading to amputation, which seriously endangers people’s physical and mental health [20]. The disease is mainly caused by repeated stress or other forms of foot trauma in the focal area, and is also associated with neuropathy [21, 22]. Studies have found that DFU is usually infected by multi-bacteria infection [23]. In this paper, 16S rRNA sequencing analysis showed that mixed bacterial infection would accelerate foot ulcers, and Corynebacterium accounted for a large proportion in the distribution of wound microflora. Through cell experiments, it was found that Corynebacterium could significantly inhibit cell proliferation, promote inflammation, apoptosis and pyroptosis.

Wound healing is a complex process that may be affected by various factors. Under most conditions, bacteria are highly pathogenic in the wound environment and can cause wound infection or continuous deterioration [24]. 16S rRNA sequencing is a useful tool for species identification and classification of bacteria. The application of 16S rRNA sequencing in diseases can characterize the structure and composition of microbiota in diseases, so as to diagnose and treat diseases more effectively and conveniently. Herein, 16S rRNA sequencing displayed that Corynebacterium accounted for a large proportion in the microflora of foot ulcer wounds. Through Logistics regression model analysis and Person correlation analysis, mixed microflora infection made the disease more serious, and the number of bacteria was closely related to inflammation, immunity and pyroptosis.

Pyroptosis has been shown to play a critical role in the wound healing of DFU. Excessive pyroptosis can trigger a persistent inflammatory response, leading to the occurrence of some inflammatory diseases [25]. There is evidence that pyroptosis promotes diabetic complications, including diabetes-induced wound [26]. In DFU rats, bone marrow mesenchymal stem cells can promote cell proliferation and angiogenesis, reduce pyroptosis to promote wound healing [27]. The MALAT1/miR-374a-5p signaling axis can suppress pyroptosis to contribute to the healing of DFU [28]. In addition, many microorganisms have been identified to induce pyroptosis-mediated disease progression, including *Legionella pneumophila*, *Salmonella*, *Shigella flexneri*, and *Listeria monocytogenes* [29]. Pregnancy-induced microbiota changes in the intestine lead to macrophage pyroptosis and drive septic inflammation progression [30]. Corynebacterium belongs to Gram-positive bacilli, including *C. diphiheriae*, *C. xerosis*, *C. pseudodiphtheriticum* and *C. striatum*, etc. [31]. Corynebacterium is mostly a conditional pathogen, which is generally non-pathogenic, and often exists in the nasal cavity, throat, conjunctiva and skin surface of humans or animals [32–34]. In this study, Corynebacterium was found to account for a large proportion of DFU through analysis, and Corynebacterium was proved to inhibit cell proliferation, promote inflammation, apoptosis and pyroptosis through in vitro experiments, which was similar to previous research results. The accumulation of intracellular *Staphylococcus aureus* triggers pyroptosis, prolongs inflammation and delays the healing of DFU [14]. In diabetic mice, *Candida albicans* infection can cause oxidative stress in foot wounds, and cause pyroptosis and apoptosis, which aggravates skin damage in diabetic mice [35]. It was concluded that Corynebacterium can promote pyroptosis to aggravate the development of DFU.

In summary, in DFU, combined with statistical analysis, 16S rRNA sequencing showed that mixed bacterial infection was more likely to worsen the disease, and Corynebacterium was more related to DFU progression. Through in vitro experiments, we found that *Corynebacterium striatum* can promote apoptosis and pyroptosis, so it is inferred that Corynebacterium may aggravate the progression of DFU by promoting pyroptosis, which provides a new target and direction to accelerate wound healing in DFU in the clinic. However, there are still some limitations in this study. This study did not analyze the bacterial communities in wounds at different depths, and due to defects in 16S rRNA sequencing technology, bacteria can only be identified at the genus level. Additionally, in future studies, DFU animal models should be constructed to explore the effect of Corynebacterium on DFU in vivo.

### Supplementary Information

**Supplementary Material 1. **

**Supplementary Material 2. **

## Data Availability

Sequence data that support the findings of this study have been deposited in the National Center for Biotechnology Information with the primary accession code PRJNA1068751.
