# Supplementary material for: 16S rRNA seq-identified Corynebacterium promotes pyroptosis to aggravate diabetic foot ulcer
Source: BMC Infect Dis. 2024 Apr 1;24:366. doi: 10.1186/s12879-024-09235-x (PMC10986075; doi:10.1186/s12879-024-09235-x)
Supplement: Supplementary file 1 — Supplementary Material 1. [file 12879_2024_9235_MOESM1_ESM.docx]

**16S rRNA seq-identified Corynebacterium promotes pyroptosis to accelerate diabetic foot ulcer**

**Table S1 Primer sequences used for RT-qPCR in this study**

| Name | Sense | Antisense |
| --- | --- | --- |
| GAPDH | AACTTTGGCATTGTGGAAGG | ACACATTGGGGGTAGGAACA |
| GSDMD | GGTTCTGGAAACCCCGTTAT | CCAGGTGTTAGGGTCCACAC |
| NLRP3 | CTTCTCTGATGAGGCCCAAG | GCAGCAAACTGGAAAGGAAG |
