# Supplementary figures and images for: 16S rRNA seq-identified Corynebacterium promotes pyroptosis to aggravate diabetic foot ulcer
Source: BMC Infect Dis. 2024 Apr 1;24:366. doi: 10.1186/s12879-024-09235-x (PMC10986075; doi:10.1186/s12879-024-09235-x)

Figure6B NLRP3

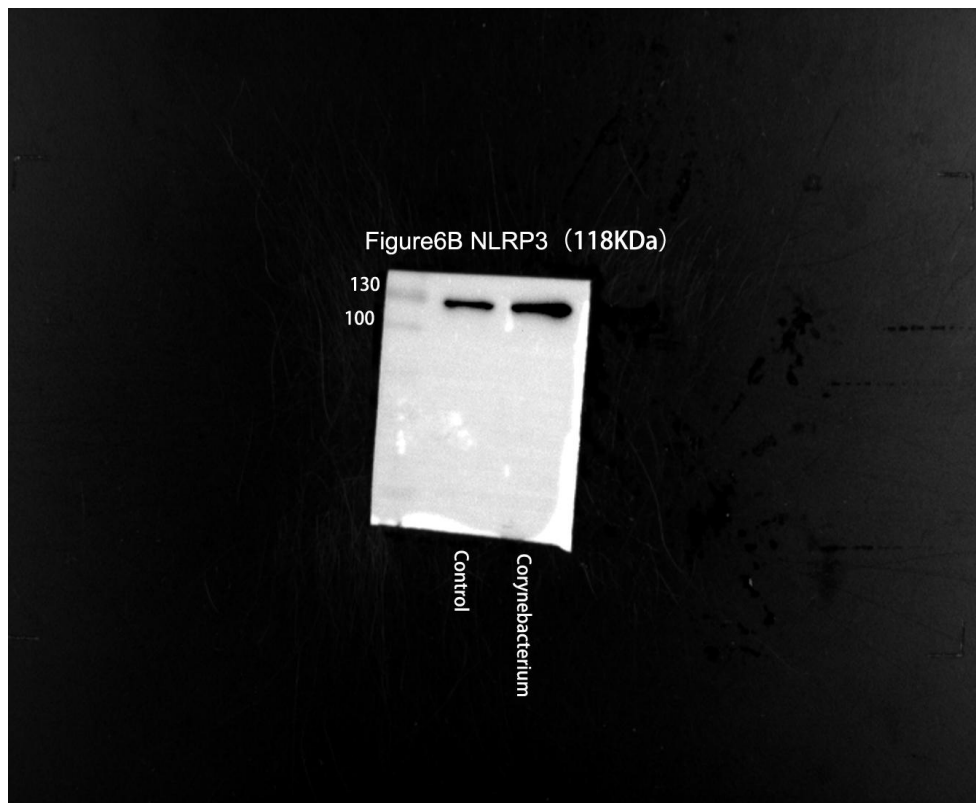

Figure6B GSDMD

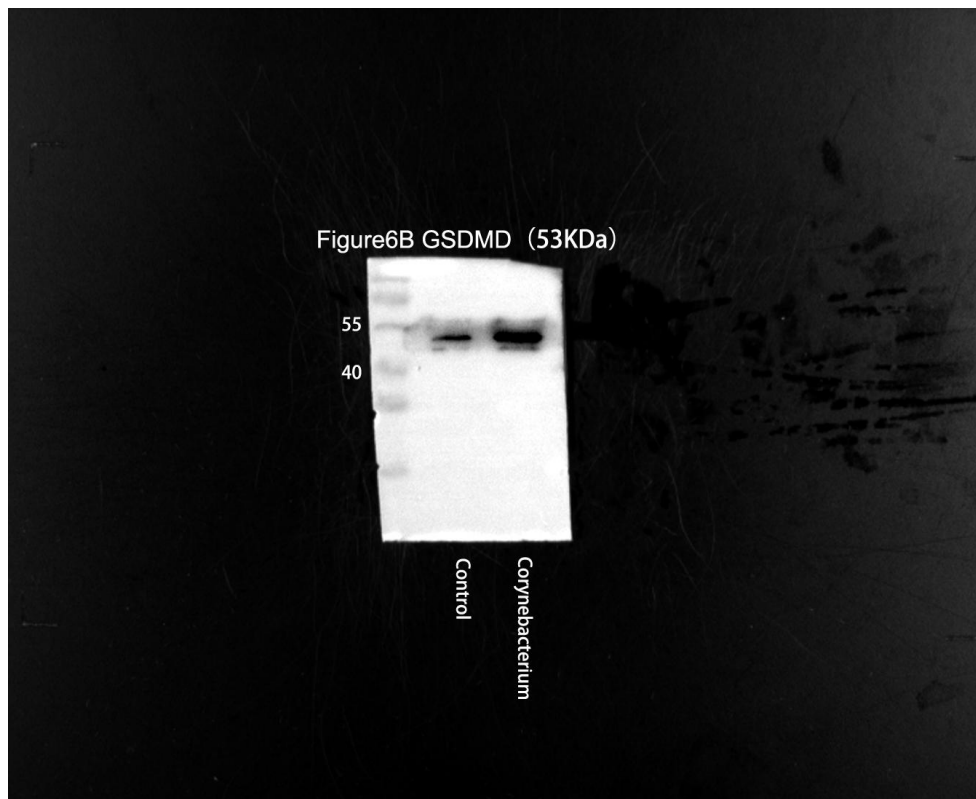

Figure6B Caspase-3

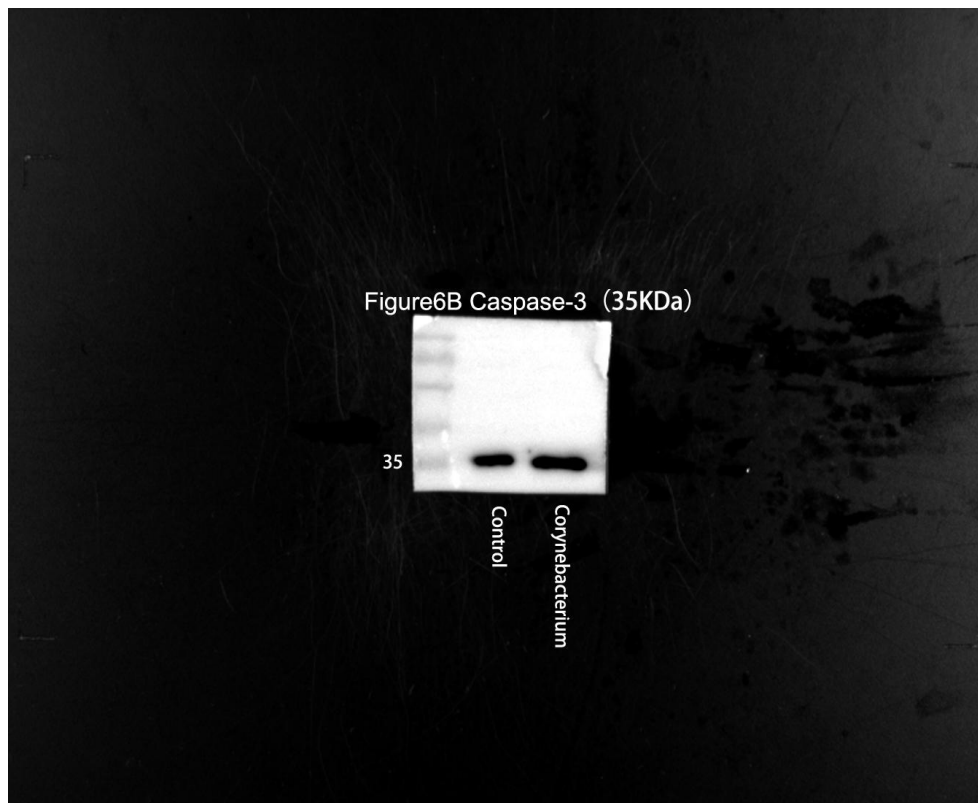

Figure6B GAPDH

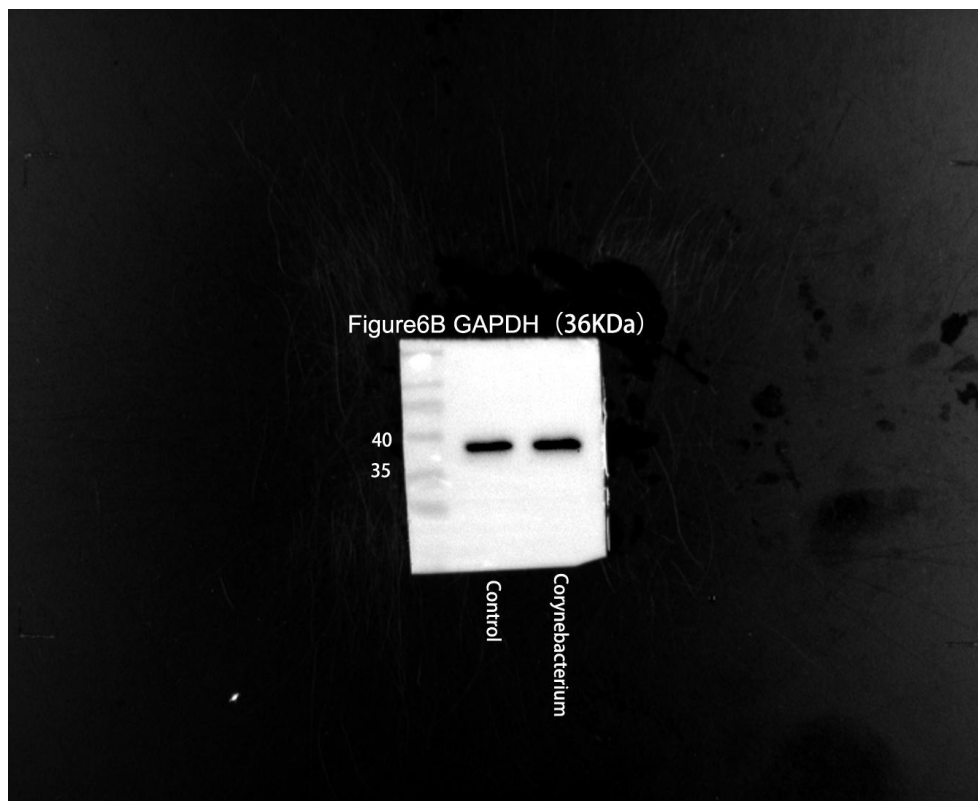

Supplement: Supplementary file 2 — Supplementary Material 2. [file 12879_2024_9235_MOESM2_ESM.pdf]
